# Long-Term Outcomes and Clinical Course of Pediatric Intestinal Pseudo-Obstruction: A Retrospective Single-Center Cohort Study

**DOI:** 10.3390/jcm15134900

**Published:** 2026-06-24

**Authors:** Kardelen Akin, Serenay Alaca, Betül Aksoy, Şenay Onbaşı Karabağ, Sinem Kahveci, Yeliz Çağan Appak, Masallah Baran

**Affiliations:** Department of Pediatric Gastroenterology, Hepatology and Nutrition, Faculty of Medicine, Izmir Katip Celebi University, İzmir City Hospital, Izmir 35620, Türkiye

**Keywords:** pediatric intestinal pseudo-obstruction, intestinal failure, gastrointestinal motility disorders, genetics

## Abstract

**Objective:** Pediatric intestinal pseudo-obstruction (PIPO) is a rare, severe, and heterogeneous gastrointestinal motility disorder associated with intestinal failure, recurrent hospitalizations, and significant morbidity and mortality. This study aimed to evaluate the clinical features, management strategies, and long-term outcomes of children diagnosed with PIPO at a tertiary referral center. **Methods:** This retrospective single-center study included pediatric patients diagnosed with PIPO between 2011 and 2025. Diagnosis was established according to ESPGHAN consensus criteria. Demographic characteristics, clinical presentation, genetic findings, nutritional support, surgical interventions, intestinal transplantation, and long-term outcomes were retrospectively reviewed. **Results:** A total of 32 patients with PIPO were included, of whom 56.2% were female and 43.7% had early-onset disease. Genetic testing was performed in 22 of 32 patients; clinically significant variants were identified in 16 (50% of the total cohort), most commonly ACTG2 mutations. Prior abdominal surgery before referral was present in 84.3% of patients. During follow-up, 56% remained parenteral nutrition dependent, five patients underwent intestinal transplantation, and the overall mortality rate was 21.8%. **Conclusions:** PIPO is a highly heterogeneous disorder associated with substantial morbidity, prolonged nutritional support requirements, repeated surgical interventions, and significant mortality. Early diagnosis, genetic evaluation, multidisciplinary management, and timely referral to specialized intestinal failure and transplantation centres are likely to support more individualised management and may help prevent avoidable complications in affected children.

## 1. Introduction

Paediatric intestinal pseudo-obstruction (PIPO) is a rare but severe gastrointestinal motility disorder characterised by recurrent or chronic symptoms of intestinal obstruction in the absence of a mechanical cause. Most cases of PIPO are congenital and become clinically evident within the first months of life; however, disease onset may range from infancy to young adulthood. Clinical manifestations vary depending on the severity and anatomical extent of gastrointestinal involvement. In the neonatal period, vomiting, abdominal distension, and delayed passage of meconium are common presenting features, whereas older children more frequently present with chronic constipation, enteral feeding intolerance, and growth failure. Prenatal warning signs, such as megacystis or dilated bowel loops on ultrasonography, have been reported in approximately 20% of affected patients [1]. Diagnosis of PIPO is primarily clinical and requires careful exclusion of mechanical obstruction. Radiological investigations, antroduodenal manometry, and, when indicated, full-thickness intestinal biopsies play an important role in the diagnostic evaluation.

From an aetiological perspective, PIPO is classified into three main categories: primary, secondary, and idiopathic. Primary PIPO accounts for approximately 80% of cases and includes neuropathic, myopathic, and mesenchymopathic forms caused by abnormalities of the enteric nervous system, intestinal smooth muscle, or interstitial cells of Cajal. Secondary PIPO may occur in association with a wide range of systemic disorders, including connective tissue diseases, mitochondrial disorders, endocrinopathies, and neurological conditions [2].

The clinical course of the disease is highly variable. While some patients can be managed conservatively with medical treatment, others develop severe disease requiring long-term parenteral nutrition (PN), prolonged hospitalisation, or intestinal transplantation (ITx). Therefore, identification of the underlying aetiology—particularly through genetic evaluation—may play a critical role in prognosis, treatment planning, and long-term follow-up [3]. Consequently, the relationship between underlying genetic mutations and disease prognosis remains an area of ongoing interest. Accordingly, this study aimed to describe the clinical spectrum of PIPO, with particular emphasis on underlying genetic findings and the diversity of disease course, nutritional requirements, and long-term follow-up strategies in affected children.

## 2. Materials and Methods

This retrospective descriptive study included patients diagnosed with PIPO and followed at our tertiary referral centre between 2011 and 2025. PIPO was diagnosed according to the diagnostic criteria proposed by European Society for Paediatric Gastroenterology, Hepatology and Nutrition (ESPGHAN). In line with these recommendations, PIPO should be considered in children who exhibit persistent or recurrent obstructive symptoms lasting at least two months from birth or at least six months thereafter. After exclusion of mechanical obstruction, patients fulfilling at least two of the following four major criteria were included [4]:

1. Objective evidence of small intestinal neuromuscular involvement (including manometry, histopathology, or transit studies),
2. Recurrent and/or persistent intestinal dilatation,
3. Presence of a genetic and/or metabolic disorder associated with PIPO, and
4. Inability to maintain adequate growth and nutrition with oral feeding, necessitating enteral and/or parenteral nutritional support.

Patients who did not meet these criteria were excluded. A total of 32 patients who fulfilled the diagnostic criteria were included in the study. Medical records were reviewed retrospectively, and data regarding demographic characteristics, clinical course, and diagnostic processes were collected for each patient. Evaluated variables included demographic characteristics such as age, sex, age at diagnosis, presence of gastrostomy or ileostomy, and presence of an affected sibling. In addition, data regarding PN requirements—including age at initiation, duration of therapy, long-term dependence, and survival time—were recorded. To assess growth and nutritional status, weight and height measurements obtained at presentation and during follow-up were used to calculate z-scores [5], using WHO and national growth reference standards with a standardised Z-score calculator.

Patients were further categorised into early-onset and late-onset groups according to the age at symptom onset. Patients who developed symptoms within the first month of life were classified as having early-onset disease, whereas those with symptom onset after the first month were classified as having late-onset disease [4]. All demographic, clinical, nutritional, and growth-related variables were analysed separately for these two groups.

Medical treatment strategies, including prokinetic agents and other supportive therapies, were reviewed. In patients requiring surgical intervention, details of the procedures performed were documented. Furthermore, advanced genetic investigations, including panel testing, identified genetic variants, and genotype–phenotype correlations were analysed. All data were obtained through a comprehensive review of patient charts, laboratory results, radiological records, and genetic reports. Genetic investigations were performed using targeted gene panel testing covering genes associated with smooth muscle and enteric nervous system disorders (including, but not limited to, ACTG2 and MYH11). Given the extended inclusion period (2011–2025), it is acknowledged that the composition of available gene panels and testing strategies may have evolved over time, which may have influenced diagnostic yield.

Patients’ nutritional status was classified according to the definitions of the European Society for Clinical Nutrition and Metabolism (ESPEN). Total parenteral nutrition (TPN) was defined as the intravenous administration of all essential nutrients required to meet the metabolic needs of patients unable to obtain adequate nutrition via the gastrointestinal tract. Patients receiving 30–80% of their nutritional requirements via the parenteral route were classified as receiving partial parenteral nutrition (PPN). Patients receiving ≥25% but <75% of their estimated caloric needs via the enteral route were classified as receiving partial enteral nutrition (PEN). In cases where both enteral and parenteral nutrition were administered, classification was based on the predominant route of nutritional support [6].

Intestinal failure-associated liver disease (IFALD) was defined after exclusion of alternative causes of liver disease. The diagnosis was based on persistent elevation of cholestatic enzymes—alkaline phosphatase (ALP) and γ-glutamyl transferase (γGT)—to >1.5 times the upper limit of normal for at least six weeks. Radiological findings were considered supportive when available, whereas liver histology was not required and was evaluated on a case-by-case basis [7].

During the preparation of this manuscript, a generative artificial intelligence tool (ChatGPT-4o, OpenAI, https://chat.openai.com)was used solely to assist with language editing, grammatical revision, and clarity of phrasing in the English text. The AI tool was not involved in data collection, analysis, interpretation, or the formulation of scientific conclusions. All content was reviewed and edited by the authors, who take full responsibility for the accuracy and integrity of the published work.

The study protocol was reviewed and approved by the Institutional Ethics Committee of our centre (approval number: 2025/139).

### Statistical Analysis

All statistical analyses were performed using Statistical Package for the Social Sciences software (version 25.0; IBM Corp., Armonk, NY, USA). Categorical variables (sex, prematurity, diagnostic classification, presence of ostomy and gastrostomy, ITx, IFALD, home PN, weaning off PN, and results of genetic analysis) were analysed using the chi-square (χ^2^) test and are presented as numbers and percentages. The Kolmogorov–Smirnov and Shapiro–Wilk tests were used to assess the normality of continuous variables. Non-normally distributed continuous variables (age, follow-up period, age at onset of symptoms, baseline and final weight, height, and body mass index standard deviation scores) were compared between groups using the Mann–Whitney U test. As most continuous variables did not follow a normal distribution, nonparametric tests (Mann–Whitney U and chi-square) were used throughout the analyses. Accordingly, the results are presented as median and interquartile range (IQR) values. The consistency of the data distribution and robustness of the comparisons were also examined. A *p*-value < 0.05 was considered statistically significant.

Survival analysis was performed using the Kaplan–Meier method. Time to event was defined as the follow-up duration from baseline to death. Patients who were alive at the last clinical contact were treated as censored observations. The log-rank (Mantel–Cox) test was used to compare survival distributions between the early- and late-onset groups. Sensitivity analyses using the Breslow (generalised Wilcoxon) and Tarone–Ware tests were also performed.

## 3. Results

A total of 32 patients with PIPO were included in the analysis. Of these, 56% (n = 18) were female, and 43.7% (n = 14) had early-onset disease. Among patients with late-onset disease, the median age at symptom onset was 102 months (range: 42–157). The majority of patients were born at term (90.6%), and genetic testing was performed in 22 of 32 patients, of whom 16 had a clinically significant variant identified (50% of the total cohort). A positive family history of PIPO in a sibling was present in four patients (12.5%). In addition, 84.3% of patients had undergone abdominal surgery prior to their initial consultation at our centre. Additional demographic and clinical characteristics are summarised in Table 1.

Two patients with secondary PIPO were also included in the cohort. Both had been previously healthy until 11 and 14 years of age, respectively, with no prior history of intestinal motility disorders. In the first patient, acute axonal sensorimotor polyneuropathy at 14 years of age was followed by progressive dysphagia and subsequent loss of intestinal motility. This patient was managed with gastrostomy and total parenteral nutrition but died during follow-up due to sepsis at 19 years of age. The second patient, aged 11 years, developed swallowing dysfunction and inability to defecate following surgery for a paravertebral ganglioneuroma. Peroral endoscopic myotomy was performed for an achalasia-like clinical presentation, and the patient has been followed for five years with ileostomy, gastrostomy, and partial home PN.

Genetic analysis was performed in 22 of the 32 patients included in the cohort. Clinically significant genetic variants associated with PIPO were identified in 16 patients (72.7% of those tested, representing 50% of the total cohort). Consanguinity was present in 46.8% of the total cohort (n = 15/32). A history of PIPO in a sibling was documented in four patients (12.5%), including two pairs of affected siblings (two female and two male). Molecular analyses revealed pathogenic or clinically significant variants in the ACTG2 gene in nine patients and in the MYH11 gene in two patients. In addition to these primary PIPO-associated variants, several syndromic conditions known to be associated with gastrointestinal motility disorders were identified, including one patient with enteric anendocrinosis (EA), three patients with Waardenburg syndrome (WS), and one patient with neurofibromatosis type 1 (NF-1). The genetic findings and corresponding clinical characteristics of the patients are summarised in Table 2.

The median follow-up duration for patients at our centre was 12 months (Q1:5–Q3:57 months). During follow-up, nine patients were successfully weaned from PN-, corresponding to a PN discontinuation rate of 33.3% (n = 9). A total of nine patients were diagnosed with IFALD. Of these, two patients who had been listed for multivisceral transplantation died due to sepsis. The frequencies of ileostomy and gastrostomy placement, as well as anthropometric measurements, are presented in Table 3.

ITx was performed in five patients with PIPO. The first patient, a male who underwent ITx at nine months of age, developed post-transplant lymphoproliferative disease (PTLD) during the first year after transplantation and died of sepsis at 21 months of age. The second patient underwent ITx at two years of age and was subsequently referred to another centre for planned multivisceral transplantation due to early graft dysfunction and concomitant need for liver transplantation. The third patient remains under follow-up 10 years after transplantation, with an ileostomy in place and maintained on full enteral nutrition. The fourth patient, a female who underwent ITx at six years of age, died due to disseminated intravascular coagulation (DIC) in the early postoperative period.

The fifth patient was an 11-year-old girl who developed short bowel syndrome secondary to malrotation associated with PIPO. She died of sepsis two months after ITx.

**Figure 1:**
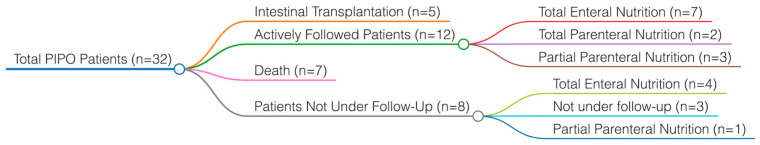
Distribution of long-term clinical outcomes and nutritional status in 32 patients with PIPO.

Of the 32 patients with PIPO, eight were lost to follow-up over time. At present, 13 patients, including one with a history of small bowel transplantation, continue to receive follow-up and treatment at our centre (Figure 1). Patients lost to follow-up were treated as censored observations in the survival analysis.

**Figure 2:**
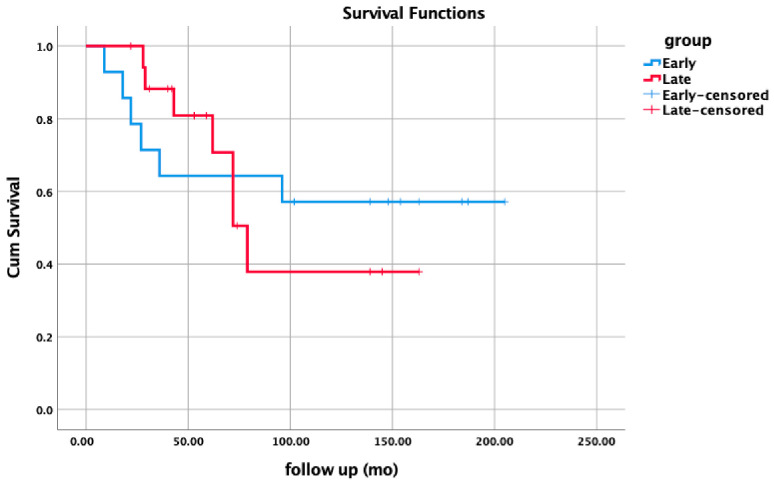
Kaplan–Meier survival curves according to study groups. Plus symbols (+) indicate censored observations.

Of the 32 patients included in the survival analysis, four of the 14 in the early-onset group and seven of the 18 in the late-onset group died during follow-up. Kaplan–Meier analysis revealed a higher cumulative survival probability for the early-onset group than in the late-onset group; however, this difference did not reach statistical significance (log-rank test: χ^2^ = 2.647, *p* = 0.104). Similar findings were observed with the Breslow (generalised Wilcoxon) and Tarone–Ware tests, further supporting the absence of a statistically significant difference between the groups (Figure 2).

## 4. Discussion

PIPO represents a highly heterogeneous clinical entity with a broad spectrum of disease severity. In our 14-year cohort, the clinical course varied markedly among patients. While some patients were managed conservatively with medical therapy, others developed severe disease requiring long-term PN, prolonged hospital dependence, or ITx. Identification of the underlying aetiology—particularly through genetic evaluation—is therefore critical for prognosis, treatment planning, and long-term follow-up. Improved understanding of genotype–phenotype correlations may further clarify disease mechanisms and support more individualised management strategies in patients with PIPO.

Malnutrition in PIPO may be illness-related (secondary to inflammation, infection, or dysmotility), non-illness related (due to environmental or behavioural factors), or multifactorial [8]. Furthermore, impaired gastrointestinal motility predisposes patients to bacterial overgrowth, which may further compromise nutrient absorption [8]. In our cohort, 34% of patients had a body weight below −3 SDS at presentation, whereas this rate decreased to 25% at the last follow-up evaluation, suggesting partial improvement in nutritional status over time. In contrast, 22% of patients had a height below −3 SDS at presentation, and this proportion remained unchanged at the last follow-up, indicating persistent impairment in linear growth.

Among the genes associated with PIPO, *ACTG2* is the most frequently identified genetic cause. Consistent with previous reports, *ACTG2* mutations were the most common genetic variants detected in our cohort, accounting for 40% of patients who underwent genetic testing [9]. Although *ACTG2*-related disease is typically inherited in an autosomal dominant manner, many cases arise from de novo variants. Pranjali et al. reported that approximately 73% of *ACTG2* mutations occur de novo [10]. In our cohort, three patients had a positive family history of PIPO among first-degree relatives; however, detailed parental segregation analyses were unavailable, limiting definitive conclusions regarding inheritance patterns in these families.

While *ACTG2* mutations are the most commonly identified genetic cause of PIPO, the relationship between genotype and clinical phenotype remains incompletely understood [10,11]. This heterogeneity was also observed in two affected brothers carrying the same *ACTG2* mutation. While the older sibling had no history of surgical intervention and demonstrated marked clinical improvement with prucalopride therapy, the younger sibling experienced a more severe disease course, required surgical intervention, and did not demonstrate a comparable response to the same treatment. Nevertheless, both patients remained free of PN throughout follow-up and did not require ostomy or gastrostomy.

Among the genetic conditions associated with PIPO in our cohort, Waardenburg syndrome was the second most frequently identified condition and was observed in three patients. Previous reports have described patients with Waardenburg syndrome presenting either with aganglionic Hirschsprung disease or with isolated gastrointestinal motility [12]. In addition, *MYH11* mutations were identified in two patients, representing one of the most frequently reported genetic alterations after *ACTG2* [13]. *MYH11* is among the cytoskeletal protein genes implicated in the pathogenesis of myopathic PIPO, and both autosomal dominant and autosomal recessive inheritance patterns have been described. Moreover, specific variants have been associated with severe phenotypes such as megacystis–microcolon–intestinal hypoperistalsis syndrome (MMIHS) [14]. In our cohort, one patient demonstrated clinical findings compatible with this phenotype [15].

Previous studies have reported considerable variability in PN dependence among patients with PIPO. A study conducted in Korea reported a PN dependence rate of approximately 24%, whereas Mousa et al. described substantially higher rates ranging from 60% to 80% [16,17]. In another study, approximately one-third of patients were enrolled in a home PN programme [18]. In our cohort, 56% of patients were PN dependent, of whom 15.6% (n = 5) were managed with home PN. In contrast, 16% of patients were maintained on total enteral nutrition during follow-up.

The relatively high rate of PN dependence observed in our cohort may be attributed to multiple factors, including delayed diagnosis resulting in malnutrition, previous surgical burden, recurrent infections, nutritional status at presentation, and limited access to or prolonged waiting times for ITx in patients with severe disease. Gastrointestinal drainage may provide symptomatic relief and prevent complications of chronic bowel dilatation, such as ischaemia and perforation [19,20]. In our cohort, both medical management and surgical interventions, including enterostomy and gastrostomy, were associated with successful weaning from PN; however, given the retrospective design, causality cannot be established and these associations should be interpreted with caution. Two patients were successfully weaned from PN following ITx, while two others achieved PN independence after tolerating enteral nutrition following ileostomy in combination with medical therapy. Among the four patients who were initially dependent on total PN, enterostomy enabled transition to partial PN at home.

Furthermore, gastrostomy and enterostomy reduced abdominal distension and vomiting during follow-up, facilitated increased enteral intake, and enabled partial home-based management of exacerbations, thereby reducing the need for hospitalisation [17]. Previous studies have demonstrated that improved outcomes and greater utilisation of home PN are associated with well-structured healthcare systems, specialised intestinal failure centres, and multidisciplinary teams [20]. In our setting, the limited availability of structured home PN programmes may have contributed to the lower utilisation rates observed in our cohort and may also have influenced long-term nutritional outcomes.

In both adults and children with PIPO, various prokinetic agents have been used to improve gastrointestinal motility and control symptoms such as nausea and vomiting. However, their use in paediatric patients remains limited due to variable clinical efficacy and the risk of significant extra-intestinal side effects [4]. In our cohort, four patients were successfully weaned from PN with medical therapy alone, without the need for venting ostomy. Additionally, improvement in abdominal distension and vomiting was observed in three patients managed on an outpatient basis.

Previous clinical studies have shown that repeated abdominal surgeries are associated with increased morbidity and mortality in patients with PIPO, and current guidelines recommend avoiding unnecessary or repeated surgical interventions whenever possible [4,21,22]. In our cohort, 84.7% of patients had already undergone surgical intervention at the time of diagnosis, and 44% had a history of three or more abdominal surgeries during follow-up. Notably, one patient who had undergone more than 30 abdominal operations was referred to our centre with an open abdomen and was subsequently evaluated as a candidate for multivisceral transplantation. In addition, one patient with PIPO associated with intestinal malrotation developed short bowel syndrome and subsequently underwent ITx. Another patient developed short bowel syndrome following multiple surgical interventions and later required a bowel-lengthening procedure. These findings suggest that a greater number of surgical interventions may be associated with poorer clinical outcomes. Therefore, careful patient selection and a multidisciplinary approach are essential to minimise unnecessary surgeries in patients with PIPO.

The high proportion of patients who had undergone prior abdominal surgery before referral (84.3%) highlights the ongoing diagnostic challenges associated with PIPO. Before the correct diagnosis is established, many patients are evaluated for, and sometimes surgically treated for, mechanical causes of intestinal obstruction, including intussusception and malrotation. Distinguishing PIPO from common obstructive conditions can be particularly challenging in less-experienced settings, and requires careful integration of clinical, radiological, and manometric findings [23]. Delayed recognition of the underlying diagnosis may contribute to repeated and potentially avoidable surgical interventions, further increasing disease-related morbidity.

ITx remains the only curative treatment option for patients with PIPO. According to intestinal transplant registry data, children with motility disorders constitute the second largest group requiring ITx, accounting for approximately 18% of cases [4]. In recent years, outcomes have improved considerably, with five-year survival rates approaching 70%, largely due to advances in surgical techniques, immunosuppressive therapies, and postoperative care [24]. Nevertheless, the decision to proceed with ITx should be made by an experienced multidisciplinary team. In our cohort, the transplantation rate was 15.6% (n = 5).

The finding of no statistically significant difference in survival rates between the early- and late-onset groups is consistent with the current literature. Nham et al. (2022) reported that survival rates did not differ significantly between neonatal-onset and late-onset PIPO populations [25]. Similarly, a survey discussed by Turcotte and Faure (2022) found no statistically significant difference in mortality between neonatal- and post-neonatal-onset groups [1]. Although the early-onset group in our study demonstrated a higher cumulative survival probability, the absence of statistical significance underscores the heterogeneous nature of PIPO and the shared risk of life-threatening iatrogenic complications, including IFALD and sepsis, which remain the leading causes of death in both groups.

Reported mortality rates for PIPO in the literature range from 10% to 32%, consistent with the expected clinical spectrum of the disease [4,26]. In more recent series, mortality rates have ranged from 4.8% to 32% [4]. In our cohort, the mortality rate was 21.8%, which falls within this reported range but remains relatively high. This may be explained by the fact that our centre serves as a national referral centre for intestinal failure and transplantation, which results in the referral of patients with more severe disease phenotypes, including those with multiple prior surgeries, TPN dependence, and those listed for transplantation. These factors likely contributed to the comparatively higher mortality observed in our cohort.

It is important to acknowledge that the burden of PIPO extends beyond survival and nutritional outcomes. A recent scoping review evaluating quality of life (QoL) in children with intestinal failure found that patients consistently reported worse health-related QoL compared to healthy controls across physical, emotional, social, and school functioning domains, while caregivers experienced significantly higher rates of stress and anxiety [27]. Given that PIPO is characterised by chronic symptoms, prolonged PN dependence, and repeated surgical interventions—all recognised determinants of impaired QoL—dedicated assessment of health-related QoL and integration of mental health support within multidisciplinary teams warrants specific attention in future clinical practice and research.

Several limitations of this study should be explicitly acknowledged. The retrospective single-centre design is subject to selection and referral bias and given that our centre functions as a national referral unit for severe intestinal failure and transplantation, the cohort may overrepresent patients with more severe disease phenotypes. The small overall sample size renders the study likely underpowered for subgroup analyses, including the survival comparison between early- and late-onset groups, and these findings should therefore be interpreted with caution. Genetic testing was not performed or results were unavailable in 10 of 32 patients, which may have introduced bias in the reported genetic frequencies. The median follow-up duration of 12 months (IQR 5–57 months) is relatively short in the context of a chronic condition such as PIPO, and the term ‘long-term outcomes’ in the title should be read with this limitation in mind. Eight patients were lost to follow-up, which may affect estimates of long-term PN dependence and survival. Finally, multivariable modelling was not attempted owing to the small sample size, limiting the ability to identify independent predictors of outcome.

In conclusion, our findings highlight the marked clinical heterogeneity of PIPO and emphasise the importance of individualised, multidisciplinary management. Early recognition, genetic evaluation, careful nutritional support, avoidance of unnecessary surgical interventions, and timely referral to specialised intestinal failure and transplantation centres are likely to support more individualised management and may help prevent avoidable complications in this complex patient population.

## Figures and Tables

**Table 1:** Demographic and Clinical Characteristics of Patients with PIPO (n = 32).

Variable	All Patients (n = 32)	Early-Onset (n = 14)	Late-Onset (n = 18)
Sex (Female), n (%)	18 (56.2)	8 (57.1)	10 (55.5)
Genetic diagnosis, n (%)	16 (50)	6 (42.8)	10 (55.5)
Gestational age (preterm/term), n (%)	3 (9.3)/29 (90.6)	1 (7.1)/13 (92.9)	2 (11.1)/16 (88.9)
Age at symptom onset, months (median, Q1–Q3)	9 (0–113)	1 (0–1)	102 (42–157)
Age at referral to our centre, months (median, Q1–Q3)	58 (18–133)	19 (11–57)	128 (43–168)

**Table 2:** Patients with PIPO-associated Genetic Mutations and Clinical Outcomes.

Patient No.	Age/ Sex	Genetic Variant	Age at Symptom Onset (Months)	Gastrostomy Ileostomy	Nutritional Status	Age at Outcome	Therapeutic Approach
1	13 y/F	MYH11 het, p.Ser1926Argfs*86	<1	Yes Yes	Total parenteral nutrition	13 y/alive	Listed for transplantation
2	16 y/M	MYH11 het, c2521-76 A>C	157	No Yes	Total enteral nutrition	16 y/alive	Azithromycin + domperidone + prucalopride therapy
3	19 y/F	ACTG2 hom, c.631C>T	84	Yes Yes	Total parenteral nutrition	19 y/alive	Home TPN programme
4	16 y/F	ACTG2 hom, c.631C>T	156	No Yes	Partial parenteral nutrition	16 y/alive	Home TPN programme
5	18 y/M	ACTG2 hom, c.987G>T	168	No No	Total enteral nutrition	18 y/alive	Clinical benefit from prucalopride therapy
6	15 y/M	ACTG2 hom, c.987G>T	156	No No	Total enteral nutrition	15 y/alive	Clinical benefit from Azithromycin + domperidone therapy
7	14 y/F	ACTG2 het, c.769C>T	84	Yes No	Total parenteral nutrition	14 y/ex	Candidate for multivisceral transplantation
8	6 y/F	ACTG2 het, c.796C>T	4	Yes Yes	Total parenteral nutrition	6 y/ex 2 day after ITx	ITx
9	11y/M	ACTG2 het, c.769C>T	6	No Yes	Partial parenteral nutrition	11 y/alive	Home PN programme
10	16 y/F	ACTG2 hom, c.839A>G	156	No No	Total enteral nutrition;	16 y/alive	Operated for malrotation
11	11 y/M	ACTG2 het c.763C>T	90	No No	Total enteral nutrition	11 y/alive	Clinical benefit from prucalopride therapy
12	15 y/M	Waardenburg syndrome	<1	No Yes	Total enteral nutrition	15 y/alive	ITx at 5 y
13	21 mo/M	Waardenburg syndrome	<1	Yes Yes	Total enteral nutrition	21 mo/ex one year after ITx	ITx at 9 mo
14	9 y/F	Waardenburg syndrome	<1	No Yes	Partial parenteral nutrition	9 y/alive	Operated for short bowel Home PN programme
15	3 y/F	NF-1 C1666A>G	6	Yes Yes	Total parenteral Nutrition	3 y/ex	
16	8 mo/F	Neurogenin3 hom c.510dupG	<1	No No	Partial parenteral nutrition	8 mo/ex	

**Table 3:** Prognostic Characteristics of Patients with PIPO (n = 32).

Variable	All Patients	Early-Onset	Late-Onset	*p*-Value
IFALD, n (%)	9 (28.1)	2 (14.2)	7 (38.8)	0.101
Weaning off TPN, n (%) *	9 (33.3)	6 (42.8)	3 (23.0)	
Gastrostomy, n (%)	11 (34.4)	3 (21.4)	8 (44.4)	0.174
Ileostomy, n (%)	20 (64.5)	10 (71.4)	10 (55.6)	0.471
Weight-for-age SDS at presentation (median, Q1–Q3)	−2.25 (−3.6 to −1.41)	−2.95 (−4.0 to −1.54)	−2.06 (−3.26 to −0.53)	0.377
Weight-for-age SDS at last follow-up (median, Q1–Q3)	−2.54 (−3.36 to −1.67)	−2.6 (−3.13 to −2.12)	−1.80 (−4.09 to −0.45)	0.317
Height-for-age SDS at presentation (median, Q1–Q3)	−1.6 (−3.0 to 0.11)	−1.95 (−3.58 to −1.35)	−0.79 (−2.30 to 0.91)	0.075
Height-for-age SDS at last follow-up (median, Q1–Q3)	−1.91 (−3.57 to −0.82	−2.31 (−4.20 to −1.98)	−1.01 (−2.36 to 0.77)	0.032

* The proportion of patients weaned off TPN (33.0%) is calculated among patients who were receiving parenteral nutrition at any point during follow-up (n = 27), not the total cohort (n = 32). All other proportions are calculated using the total cohort (n = 32) as denominator unless otherwise stated. Abbreviations: IFALD, intestinal failure-associated liver disease; TPN, total parenteral nutrition; SDS, standard deviation score.

## Data Availability

The datasets supporting the conclusions of this article are included within the article.
